# Gender differences, environmental pressures, tumor characteristics, and death rate in a lung cancer cohort: a seven-years Bayesian survival analysis using cancer registry data from a contaminated area in Italy

**DOI:** 10.3389/fpubh.2023.1278416

**Published:** 2024-01-10

**Authors:** Antonia Mincuzzi, Simona Carone, Claudia Galluzzo, Margherita Tanzarella, Giovanna Maria Lagravinese, Antonella Bruni, Ivan Rashid, Lucia Bisceglia, Rodolfo Sardone, Francesco Addabbo, Sante Minerba, Orazio Valerio Giannico

**Affiliations:** ^1^Unit of Statistics and Epidemiology, Local Health Authority of Taranto, Taranto, Italy; ^2^Coordination Center of the Apulia Cancer Registry, Strategic Regional Agency for Health and Social Care of Apulia, Bari, Italy; ^3^Healthcare Management, Local Health Authority of Taranto, Taranto, Italy

**Keywords:** bronchus cancer, lung cancer, cancer survival, gender differences, environmental contamination, environmental pollution, cancer epidemiology

## Abstract

**Introduction:**

In Taranto, Southern Italy, adverse impacts on the environment and human health due to industrial installations have been studied. In the literature, associations have been reported between gender, environmental factors, and lung cancer mortality in women and men. The aim of this study was to investigate the relationships between gender, residence in areas with high environmental pressures, bronchus/lung cancer characteristics, and death rate.

**Methods:**

Data from the Taranto Cancer Registry were used, including all women and men with invasive bronchus/lung cancer diagnosed between 1 January 2016 and 31 December 2020 and with follow-up to 31 December 2022. Bayesian mixed effects logistic and Cox regression models were fitted with the approach of integrated nested Laplace approximation, adjusting for patients and disease characteristics.

**Results:**

A total of 2,535 person-years were observed. Male gender was associated with a higher prevalence of histological grade 3 (OR 2.45, 95% CrI 1.35–4.43) and lung squamous-cell carcinoma (OR 3.04, 95% CrI 1.97–4.69). Variables associated with higher death rate were male gender (HR 1.24, 95% CrI 1.07–1.43), pathological/clinical stage II (HR 2.49, 95% CrI 1.63–3.79), III (HR 3.40, 95% CrI 2.33–4.97), and IV (HR 8.21, 95% CrI 5.95–11.34), histological grade 3 (HR 1.80, 95% CrI 1.25–2.59), lung squamous-cell carcinoma (HR 1.18, 95% CrI 1.00–1.39), and small-cell lung cancer (HR 1.62, 95% CrI 1.31–1.99). Variables associated with lower death rate were other-type lung cancer (HR 0.65, 95% CrI 0.44–0.95), high immune checkpoint ligand expression (HR 0.75, 95% CrI 0.59–0.95), lung localization (HR 0.73, 95% CrI 0.62–0.86), and left localization (HR 0.85, 95% CrI 0.75–0.95).

**Discussion:**

The results among patients with lung cancer did not show an association between residence in the contaminated site of national interest (SIN) and the prevalence of the above mentioned prognostic factors, nor between residence in SIN and death rate. The findings confirmed the independent prognostic values of different lung cancer characteristics. Even after adjusting for patients and disease characteristics, male gender appeared to be associated with a higher prevalence of poorly differentiated cancer and squamous-cell carcinoma, and with an increased death rate.

## Introduction

Lung cancer is the second most diagnosed cancer and the leading cause of cancer death in 2020. With an estimated 2.2 million new cancer cases and 1.8 million deaths in 2020, it represents approximately one in 10 (11.4%) cancers diagnosed and one in 5 (18.0%) deaths (1, 2). The risk of developing this cancer is associated with older age combined with a history of smoking cigarettes. It is more common among men than women and among those with lower socioeconomic status. Among non-smokers, important lung cancer risk factors are exposure to second-hand smoke, exposure to ionizing radiation, and occupational exposure to lung carcinogens, such as asbestos (3).

Treatments for lung cancers are based on the biological subtyping of the tumors, and several disease characteristics, such as staging, grading, morphology, PD-L1 expression, topography, and laterality, represent prognostic factors and/or targets for therapies (4–9). Specifically, tumor, node, metastases (TNM) classification is a system used to describe the amount and spread of cancer in a patient’s body. In TNM classification, T describes the size of the tumor and any spread of cancer to nearby tissues, N describes the spread of cancer to nearby lymph nodes, and M describes the metastasis, i.e., the spread of cancer to other parts of the body. TNM combinations are grouped into five less-detailed stages, from 0 (carcinoma *in situ*, where abnormal cells are present but have not spread to nearby tissues) to I-II-III (invasive cancer, where the higher the number, the larger the tumor and the more it has spread to nearby tissues) to IV (invasive, metastatic cancer, where cancer has spread to distant parts of the body) (4, 10–12). In addition to TNM staging, histologic grading is a predictor of disease outcome in lung cancer patients, with higher tumor grade (lower differentiation) being associated with a poorer prognosis (differentiation describes how much a tumor resembles the normal tissue from which it arose) (5, 13). Tumor morphology is another prognostic factor in patients with lung cancer. Non-small-cell lung cancer (NSCLC) is any type of epithelial lung cancer other than small-cell lung cancer (SCLC). The most common types of NSCLC are lung adenocarcinoma (LAC) and lung squamous-cell carcinoma (LSCC), but there are several other types that occur less frequently, and all types can occur in unusual histological variants. NSCLC is usually less sensitive to chemotherapy and radiation therapy than SCLC, but patients with resectable cancer may be cured through surgery or surgery followed by chemotherapy. Conversely, SCLC is a distinct subtype of lung cancer that presents as a proliferation of small cells. It is more responsive to chemotherapy and radiation therapy than other cell types of lung cancer; however, it is difficult to cure as SCLC has a greater tendency to spread widely even before diagnosis takes place (4, 6, 7). Programmed death-ligand 1 (PD-L1) expression is also an important prognostic factor in lung cancer. PD-L1 is a ligand of the programmed death protein 1 (PD-1) coinhibitory immune checkpoint expressed on tumor cells and infiltrating immune cells. Tumors with the expression of PD-L1 ≥ 50% are amenable to first-line treatment with targeted biological agents such as pembrolizumab, with improvement in survival (4, 6, 7). In previous studies, authors have also found associations between bronchus/lung cancer topography, laterality, and death rate. Specifically, increased death rates were reported in patients with cancer localization in the main bronchus or on the right side (8, 9).

As far as gender differences are concerned, higher lung cancer incidence and mortality were reported in men, even when other clinical and demographic characteristics were considered. Regarding environmental pressures, which can also be linked to gender differences in exposure patterns, air pollution is a recognized risk factor for lung cancer incidence and mortality and is a major health concern for Europeans, with more than 300,000 premature deaths each year attributed to chronic exposure to fine particulate matter alone. Part of this mortality is due to lung cancer, and the International Agency for Research on Cancer (IARC) has classified outdoor air pollution and particulate matter in outdoor air pollution as carcinogenic to humans (Group 1), with sufficient evidence for lung cancer (14–19).

In some areas of the province of Taranto, a coastal city in the Apulia region in Southern Italy, various industrial installations and polluting sources (a steel plant, an oil refinery, urban discharges, harbor activities, and the shipyard of the Italian Navy) have been operating in close proximity to the resident population for decades with well-known and extensively studied adverse impacts on the environment and human health (20–34). With regard to environmental, feed, and food impacts, it is of particular importance that the area shows contamination of these matrices by metals and persistent organic pollutants, specifically dioxins and PCBs. Moreover, some of these substances have been detected in in human biological samples (21, 24–29). As far as human health effects are concerned, evidence has been produced after studying the populations who resided in the contaminated site of national interest (SIN) of Taranto. In particular, cohort studies have reported an increased risk for different types of cancer incidence, including lung cancer incidence in women and men (20, 31). Some studies have also noted an increased risk for all-cause hospitalization; for circulatory, respiratory, digestive, and urinary diseases hospitalization; and for different types of cancer hospitalization, including lung cancer hospitalization in women and men (20, 33, 34). Different studies have also indicated an increased risk for all-cause mortality; for circulatory and digestive diseases mortality; and for some types of cancer mortality, including lung cancer mortality in women and men (20, 23, 30, 33, 34).

To summarize, associations have been reported between the aforementioned factors and lung cancer mortality in women and men. The aim of this study was to investigate the relationships between gender, residence in areas with high environmental pressures, bronchus/lung cancer characteristics, and death rate.

## Methods

### Study area and baseline epidemiological data

**Figure 1:**
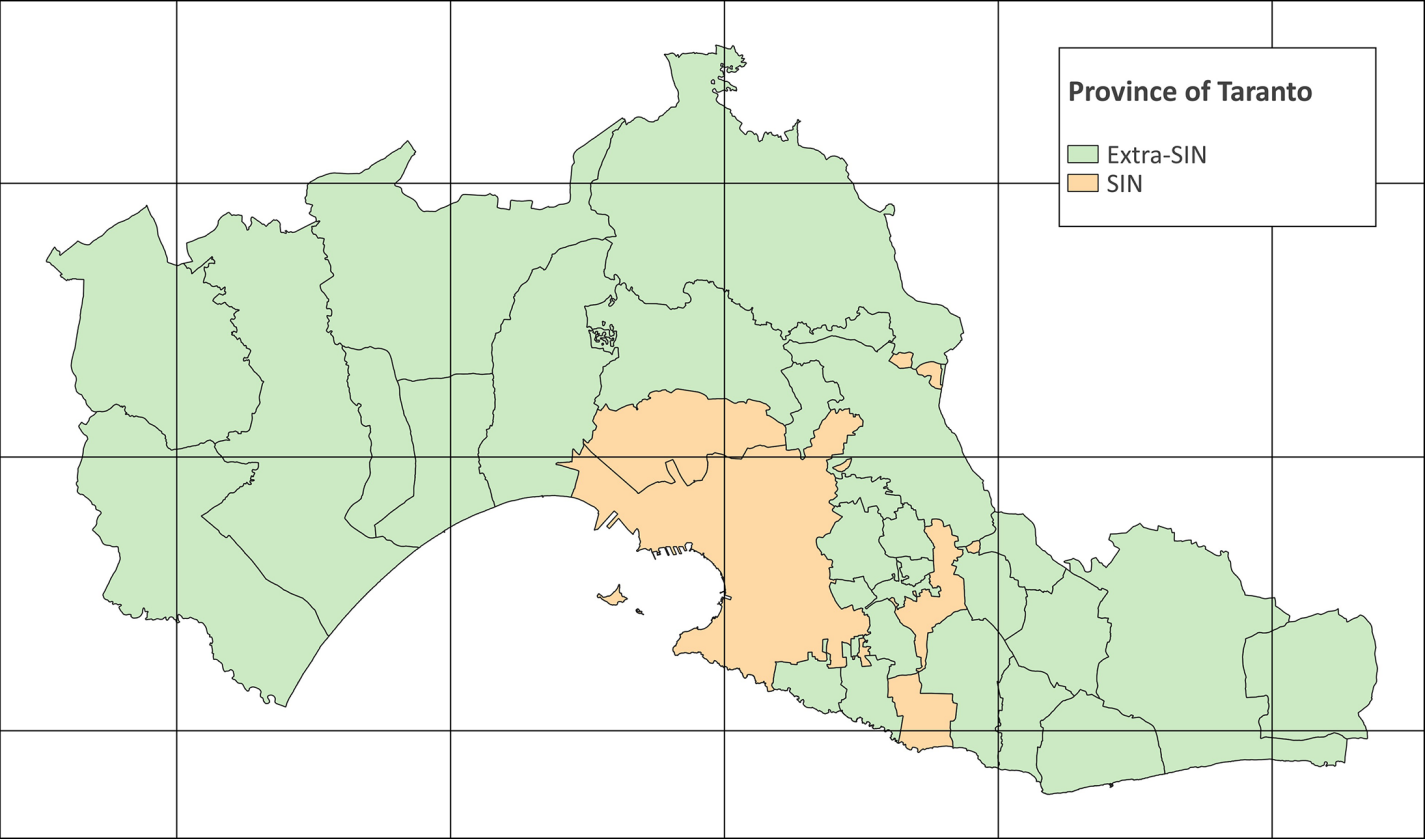
Map of the province of Taranto (grid interval: 20 km) (EPSG:32632 – WGS 84 / UTM zone 32N) (Modified from Italian National Institute of Statistics. Administrative boundaries. https://www.istat.it/it/archivio/222527).

The study area is the province of Taranto, which consists of 29 municipalities with a total resident population of 555,999 on 1 January 2023 (35). The SIN of Taranto consists of two municipalities, Taranto (the provincial capital) and Statte, respectively, with a population size of 188,098 and 12,917 on 1 January 2023 (33–35). The study area with municipalities and SIN is shown in Figure 1. The map was created with QGIS version 3.28.4.

From 2015 to 2019, Taranto Province recorded 1,740 bronchus/lung cancer (ICD10 codes C34.0 to C34.9) cases, with a directly standardized rate of 21.5 cases ber 100,000 inhabitants in women and 96.8 cases per 100,000 in men, and a median age of 70 years in women and 72 years in men. In the studied period, no trachea cancer (C33) cases were recorded. In the same period, 67% of patients with bronchus/lung cancer requiring hospitalization were admitted to a hospital in the Taranto Province, 21% to an extra-provincial hospital in the Apulia region, and 12% to an extra-regional hospital. Between 2013 and 2017, the relative standardized five-years bronchus/lung cancer survival rate was 24.5 (95% CI 19.4–29.9) for women and 17.8 (95% CI 14.7–21.2) for men (31).

From 2013 to 2017, in the SIN, 121 bronchus/lung cancer deaths were recorded among women, with a standardized mortality ratio (reference: Apulia region) of 125 (90% CI 107–145); and 451 bronchus/lung cancer deaths were recorded among men, with a standardized mortality ratio (reference: Apulia region) of 118 (90% CI 109–127) (33). From 2015 to 2019, in the SIN, 202 bronchus/lung cancer cases were recorded among women, with a directly standardized rate of 30.6 cases per 100,000 inhabitants and a standardized incidence ratio (reference: Taranto Province) of 191.2 (95% CI 165.8–219.5). For the same period, in the SIN, 582 bronchus/lung cancer cases were recorded among men, with a directly standardized rate of 111.1 cases per 100.000 inhabitants and a standardized incidence ratio (reference: Taranto Province) of 125.7 (95% CI 115.7–136.4) (31).

### Data source and cancer cohort

Data from the Taranto Cancer Registry of the Italian Association of Cancer Registries (AIRTUM) were used, including all women and men with invasive bronchus/lung cancer (ICD10 codes C34.0 to C34.9) diagnosed between 01 January 2016 and 31 December 2020 who resided in Taranto Province at the time of diagnosis. In the studied period, no cases of trachea cancer (C33) were recorded. For simplicity reasons, in this study, we refer to main bronchus (C34.0) and lung (C34.1 to C34.8) cancers as “lung cancer.” The follow-up period considered for this study was until 31 December 2022. Death certificate only cases (*n* = 23), cases registered based on an autopsy report (*n* = 1), and patients under 40 years (*n* = 4) were excluded. As a general rule, baseline patients and disease characteristics refer to the time of diagnosis. Mortality data (all-cause mortality) relative to the follow-up period (2016–2022) were retrieved from the Taranto Province’s Causes of Death and Health Registries. Patients with no mortality follow-up information due to extra-provincial transfer before 31 December 2022 (right-censoring, loss-to-follow-up) contributed to the person-time until the date of transfer (*n* = 8).

### Study design and variables

This is a retrospective individual observational study with different regression analyses carried out cross-sectionally (prevalence study) and longitudinally (incidence study, survival analysis). Residence in the areas with high environmental pressures (SIN) was used as an environmental exposure proxy. Tumor characteristics at the time of diagnosis were pathological/clinical staging (TNM I to IV), histological grading (grade 1 to 3), morphology (ICDO3M classification. LAC: Lung Adenocarcinoma; LSCC: Lung Squamous-Cell Carcinoma; SCLC: Small-Cell Lung Cancer; OTLC: Other-Type Lung Cancer; OTLC included morphologies with too low numbers for separate analysis and not included in previous groups, i.e., adenosquamous, large-cell neuroendocrine, lymphoepithelial, pleomorphic, mucoepidermoid, and pseudosarcomatous tumors, as well as different types of sarcoma) immune checkpoint ligand expression (PD-L1%), topography (ICDO3T classification. C34.0: main bronchus; C34.1: upper lobe, lung; C34.2: middle lobe, lung; C34.3: lower lobe, lung; and C34.8: overlapping lesion, lung), and laterality (unilateral right, unilateral left, and synchronous bilateral lung cancer).

In the cross-sectional study, the studied outcomes were each of the tumor characteristics (prevalence), and the studied exposures were gender and residence in areas with high environmental pressures. The aim of this step was to assess possible associations between gender, environmental factors, and each of the tumor characteristics. In the longitudinal study, the studied outcome was all-cause death (incidence), and the studied exposures were gender, residence in areas with high environmental pressures, and tumor characteristics. The aim of this step was to assess possible associations between gender, environmental factors, tumor characteristics, and death. Adjustment variables recorded at the time of diagnosis were age class (40–59, 60–69, 70–79, ≥ 80 years), year, patient ID, and municipality of residence. Adjustment for the patient’s ID and municipality of residence was provided to account for the heterogeneity related to possible unobserved individual or ecological level variables (e.g., tobacco use, alcohol consumption, social and material deprivation and access to health services).

### Statistical analysis

Data analysis was performed using R version 4.2.3. Bayesian inference was performed with package INLA version 22.12.16. Complex models could be fitted with the Bayesian approach, including generalized linear models and survival analyses. The possible non-independence and heterogeneity of observations could be taken into account by fitting mixed models with both fixed and random effects. While traditional survival analysis relies on parameter estimation based on partial likelihood, Bayesian approaches for time-to-event data allow us to use the full likelihood to estimate all unknown elements in the model. Bayesian generalized linear models comprise Bayesian logistic regression for binary response data. However, the computation of the posterior and other quantities of interest in these complex models is usually much more difficult than frequentist calculations. The Integrated Nested Laplace Approximation (INLA) is a deterministic method for Bayesian calculations that applies to a wide class of models called Latent Gaussian Models. INLA provides fast and accurate approximations to the posterior marginals through a clever use of Laplace approximations and advanced numerical methods, taking computational advantage of sparse matrices. In most cases, INLA is both faster and more accurate than other methods for Bayesian computation (36–40).

The cross-sectional study analyzed the associations between gender, residence in areas with high environmental pressures, and tumor characteristics using a series of mixed effects binary logistic regressions. Pathological/clinical staging (TNM III-IV; TNM IV), histological grading (grade 3), morphology (SCLC in patients with LAC, LSCC, or SCLC; LSCC in patients with LAC or LSCC), immune checkpoint ligand expression (PD-L1 ≥ 50%), topography (lung), and laterality (left, excluding patients with bilateral cancer) were considered as outcome measures binary variables. For each regression model, records with missing values for the analyzed outcome were excluded. Gender and residence in areas with high environmental pressures were included as fixed effects binary variables. Age class and year were included as fixed effects multinominal variables. Patient ID and municipality of residence were included as random effects multinominal variables (random intercepts). Bayesian binary logistic regression models were fitted with the INLA approach for latent Gaussian models, computing odds ratios (OR) and 95% credible intervals (CrI). An independent and identically distributed random distribution was chosen for patient ID and municipality of residence (37–39).

The longitudinal study analyzed the associations between gender, residence in areas with high environmental pressures, tumor characteristics, and death using a mixed effects Cox proportional hazard regression. The time axis was the difference in days between the day of cancer diagnosis and the last day of follow-up (event or right censoring). All-cause death was considered as the outcome measure binary variable (event). The proportional hazard assumption was verified through the analysis of plotted survival curves between the different levels of the variables. Gender, residence in areas with high environmental pressures, pathological/clinical staging (TNM I, II, III, IV), histological grading (grade 1–2, 3), morphology (LAC, LSCC, SCLC, OTLC), immune checkpoint ligand expression (PD-L1 0–49%, ≥ 50%), topography (main bronchus, lung), and laterality (right, left) were included as fixed effects binary or multinominal variables. An “NA” (not available) category was created for the records with missing values for the analyzed exposures. Due to low frequency, the bilateral cancer category was merged with the NA category. Age class and year were included as fixed effects multinominal variables. Patient ID and municipality of residence were included as random effects multinominal variables (random intercepts). Bayesian Cox regression models were fitted with the INLA approach for latent Gaussian models, computing hazard ratios (HR) and 95% credible intervals (CrI). An independent and identically distributed random distribution was chosen for patient ID and municipality of residence, while a random walk model of order two was chosen for the baseline hazard function (36–40).

Generalized variance inflation factors (GVIF) were calculated to test the presence of multicollinearity in the data. Sensitivity analyses were performed by examining the extent to which the results were affected by changes in methods, models, variables, influential observations, and inclusion/exclusion criteria. Different combinations of included patients and variables were tested, and for the included variables, different collapsed categories, as well as changes in the type of estimated effects (fixed or random), were also tested. The models were iteratively refitted by excluding from the dataset each age class and year one at a time.

## Results

Baseline patients and disease characteristics are shown in Table 1. A total of 2,535 person-years were observed, 1,893 for men, 1,212 for residents in SIN, and 1,118 for deceased patients, with a median (IQR) age of 72.0 (66.0;78.2) years.

**Table 1:** Baseline patients and disease characteristics and follow-up survival status in the lung cancer cohort, by gender, residence in SIN, and survival status.

Baseline patients and disease characteristics and follow-up survival status	Lung cancer cohort						
	*N* = 1,696; person-years = 2,535						
	Women	Men	Extra-SIN	SIN	Survived	Deceased	Total
*Age*							
Age [Median (IQR)]	70.0 (62.0;76.0)	73.0 (67.0;79.0)	72.0 (66.0;79.0)	72.0 (66.0;78.0)	69.0 (63.0;74.0)	73.0 (67.0;80.0)	72.0 (66.0;78.2)
40–59 [*n* (%)]	75 (20.60%)	126 (9.46%)	102 (11.20%)	99 (12.61%)	50 (14.29%)	151 (11.22%)	201 (11.85%)
60–69 [*n* (%)]	102 (28.02%)	350 (26.28%)	248 (27.22%)	204 (25.99%)	135 (38.57%)	317 (23.55%)	452 (26.65%)
70–79 [*n* (%)]	131 (35.99%)	542 (40.69%)	349 (38.31%)	324 (41.27%)	141 (40.29%)	532 (39.52%)	673 (39.68%)
≥ 80 [*n* (%)]	56 (15.38%)	314 (23.57%)	212 (23.27%)	158 (20.13%)	24 (6.86%)	346 (25.71%)	370 (21.82%)
*Year*							
2016 [*n* (%)]	77 (21.15%)	249 (18.69%)	182 (19.98%)	144 (18.34%)	51 (14.57%)	275 (20.43%)	326 (19.22%)
2017 [*n* (%)]	71 (19.51%)	315 (23.65%)	198 (21.73%)	188 (23.95%)	62 (17.71%)	324 (24.07%)	386 (22.76%)
2018 [*n* (%)]	60 (16.48%)	260 (19.52%)	169 (18.55%)	151 (19.24%)	67 (19.14%)	253 (18.80%)	320 (18.87%)
2019 [*n* (%)]	81 (22.25%)	277 (20.80%)	193 (21.19%)	165 (21.02%)	90 (25.71%)	268 (19.91%)	358 (21.11%)
2020 [*n* (%)]	75 (20.60%)	231 (17.34%)	169 (18.55%)	137 (17.45%)	80 (22.86%)	226 (16.79%)	306 (18.04%)
*Pathological/clinical staging*							
TNM I [*n* (%)]	40 (10.99%)	134 (10.06%)	87 (9.55%)	87 (11.08%)	131 (37.43%)	43 (3.19%)	174 (10.26%)
TNM II [*n* (%)]	21 (5.77%)	69 (5.18%)	42 (4.61%)	48 (6.11%)	45 (12.86%)	45 (3.34%)	90 (5.31%)
TNM III [*n* (%)]	15 (4.12%)	97 (7.28%)	58 (6.37%)	54 (6.88%)	36 (10.29%)	76 (5.65%)	112 (6.60%)
TNM IV [*n* (%)]	149 (40.93%)	449 (33.71%)	318 (34.91%)	280 (35.67%)	40 (11.43%)	558 (41.46%)	598 (35.26%)
NA [*n* (%)]	139 (38.19%)	583 (43.77%)	406 (44.57%)	316 (40.25%)	98 (28.00%)	624 (46.36%)	722 (42.57%)
*Histological grading*							
Grade 1 [*n* (%)]	9 (2.47%)	5 (0.38%)	7 (0.77%)	7 (0.89%)	10 (2.86%)	4 (0.30%)	14 (0.83%)
Grade 2 [*n* (%)]	19 (5.22%)	51 (3.83%)	34 (3.73%)	36 (4.59%)	38 (10.86%)	32 (2.38%)	70 (4.13%)
Grade 3 [*n* (%)]	47 (12.91%)	207 (15.54%)	135 (14.82%)	119 (15.16%)	53 (15.14%)	201 (14.93%)	254 (14.98%)
NA [*n* (%)]	289 (79.40%)	1,069 (80.26%)	735 (80.68%)	623 (79.36%)	249 (71.14%)	1,109 (82.39%)	1,358 (80.07%)
*Morphology*							
LAC [*n* (%)]	183 (50.27%)	519 (38.96%)	353 (38.75%)	349 (44.46%)	211 (60.29%)	491 (36.48%)	702 (41.39%)
LSCC [*n* (%)]	28 (7.69%)	290 (21.77%)	183 (20.09%)	135 (17.20%)	79 (22.57%)	239 (17.76%)	318 (18.75%)
SCLC [*n* (%)]	33 (9.07%)	116 (8.71%)	84 (9.22%)	65 (8.28%)	8 (2.29%)	141 (10.48%)	149 (8.79%)
OTLC [*n* (%)]	25 (6.87%)	37 (2.78%)	30 (3.29%)	32 (4.08%)	34 (9.71%)	28 (2.08%)	62 (3.66%)
NA [*n* (%)]	95 (26.10%)	370 (27.78%)	261 (28.65%)	204 (25.99%)	18 (5.14%)	447 (33.21%)	465 (27.42%)
*Immune checkpoint ligand expression*							
PD-L1 0–49% [*n* (%)]	77 (21.15%)	274 (20.57%)	184 (20.20%)	167 (21.27%)	76 (21.71%)	275 (20.43%)	351 (20.70%)
PD-L1 ≥ 50% [*n* (%)]	32 (8.79%)	105 (7.88%)	65 (7.14%)	72 (9.17%)	40 (11.43%)	97 (7.21%)	137 (8.08%)
NA [*n* (%)]	255 (70.05%)	953 (71.55%)	662 (72.67%)	546 (69.55%)	234 (66.86%)	974 (72.36%)	1,208 (71.23%)
*Topography*							
Main bronchus [*n* (%)]	40 (10.99%)	164 (12.31%)	117 (12.84%)	87 (11.08%)	16 (4.57%)	188 (13.97%)	204 (12.03%)
Lung [*n* (%)]	279 (76.65%)	1,007 (75.60%)	688 (75.52%)	598 (76.18%)	320 (91.43%)	966 (71.77%)	1,286 (75.83%)
NA [*n* (%)]	45 (12.36%)	161 (12.09%)	106 (11.64%)	100 (12.74%)	14 (4.00%)	192 (14.26%)	206 (12.15%)
*Laterality*							
Right [*n* (%)]	179 (49.18%)	682 (51.20%)	459 (50.38%)	402 (51.21%)	176 (50.29%)	685 (50.89%)	861 (50.77%)
Left [*n* (%)]	143 (39.29%)	504 (37.84%)	352 (38.64%)	295 (37.58%)	157 (44.86%)	490 (36.40%)	647 (38.15%)
Bilateral [*n* (%)]	9 (2.47%)	33 (2.48%)	21 (2.31%)	21 (2.68%)	4 (1.14%)	38 (2.82%)	42 (2.48%)
NA [*n* (%)]	33 (9.07%)	113 (8.48%)	79 (8.67%)	67 (8.54%)	13 (3.71%)	133 (9.88%)	146 (8.61%)
*Gender*							
Women [*n* (%)]	364 (100.00%)	0 (0.00%)	157 (17.23%)	207 (26.37%)	109 (31.14%)	255 (18.95%)	364 (21.46%)
Men [*n* (%)]	0 (0.00%)	1,332 (100.00%)	754 (82.77%)	578 (73.63%)	241 (68.86%)	1,091 (81.05%)	1,332 (78.54%)
*Residence in SIN*							
Extra-SIN [*n* (%)]	157 (43.13%)	754 (56.61%)	911 (100.00%)	0 (0.00%)	173 (49.43%)	738 (54.83%)	911 (53.71%)
SIN [*n* (%)]	207 (56.87%)	578 (43.39%)	0 (0.00%)	785 (100.00%)	177 (50.57%)	608 (45.17%)	785 (46.29%)
*Survival status at the end of follow-up*							
Survived [*n* (%)]	109 (29.95%)	241 (18.09%)	173 (18.99%)	177 (22.55%)	350 (100.00%)	0 (0.00%)	350 (20.64%)
Deceased [*n* (%)]	255 (70.05%)	1,091 (81.91%)	738 (81.01%)	608 (77.45%)	0 (0.00%)	1,346 (100.00%)	1,346 (79.36%)
Days of follow-up [Median (IQR)]	360.0 (86.5;1,110.2)	224.0 (62.0;817.2)	217.0 (62.0;841.5)	294.0 (72.0;899.0)	1,424.0 (1,068.2;1,910.8)	153.0 (47.0;399.8)	248.0 (65.8;864.5)
Person-years [Sum]	642.6	1,892.7	1,323.1	1,212.2	1,417.8	1,117.5	2,535.3

Province of Taranto, 2016–20, follow-up to 31/12/2022. N: cohort.

The results of the mixed effects Bayesian binary logistic regression models are reported in Table 2. Mutually adjusting and adjusting for baseline age class, year, patient ID, and municipality of residence, the fixed effect variable male gender was associated with a higher prevalence of grade 3 (OR 2.45, 95% CrI 1.35–4.43) and morphology LSCC (OR 3.04, 95% CrI 1.97–4.69), while the fixed effect variable residence in SIN did not appear to be clearly associated with the prevalence of the investigated tumor characteristics.

**Table 2:** Results of the mixed effects Bayesian INLA binary logistic regression models in the lung cancer cohort, mutually adjusted and adjusted for baseline age class, year, patient ID, and municipality of residence.

Mixed effects INLA binary logistic regressions	Lung cancer cohort							
	TNM III-IV		TNM IV		Grade 3		SCLC	
	*N* = 974; *n* = 710		*N* = 974; *n* = 598		*N* = 338; *n* = 254		*N* = 1,169; *n* = 149	
Fixed effects	OR	95% CrI	OR	95% CrI	OR	95% CrI	OR	95% CrI
*Gender*								
Women	1.00	(ref)	1.00	(ref)	1.00	(ref)	1.00	(ref)
Men	0.96	0.68–1.36	0.74	0.54–1.03	2.45	1.35–4.43	0.91	0.59–1.40
*Residence in SIN*								
Extra-SIN	1.00	(ref)	1.00	(ref)	1.00	(ref)	1.00	(ref)
SIN	0.86	0.64–1.15	0.85	0.65–1.11	0.93	0.55–1.56	0.83	0.59–1.19

Mixed effects INLA binary logistic regressions	Lung cancer cohort							
	LSCC		PD-L1 ≥ 50%		Lung localization		Left localization	
	*N* = 1,020; *n* = 318		*N* = 488; *n* = 137		*N* = 1,490; *n* = 1,286		*N* = 1,508; *n* = 647	
Fixed effects	OR	95% CrI	OR	95% CrI	OR	95% CrI	OR	95% CrI
*Gender*								
Women	1.00	(ref)	1.00	(ref)	1.00	(ref)	1.00	(ref)
Men	3.04	1.97–4.69	1.03	0.63–1.69	0.86	0.59–1.26	0.89	0.69–1.15
*Residence in SIN*								
Extra-SIN	1.00	(ref)	1.00	(ref)	1.00	(ref)	1.00	(ref)
SIN	0.80	0.61–1.06	1.24	0.83–1.85	1.16	0.86–1.57	0.95	0.77–1.17

Province of Taranto, 2016–20, follow-up to 31/12/2022. Outcome (prevalence): tumor characteristic. N: prevalent cases and non-cases. n: prevalent cases.

**Figure 2:**
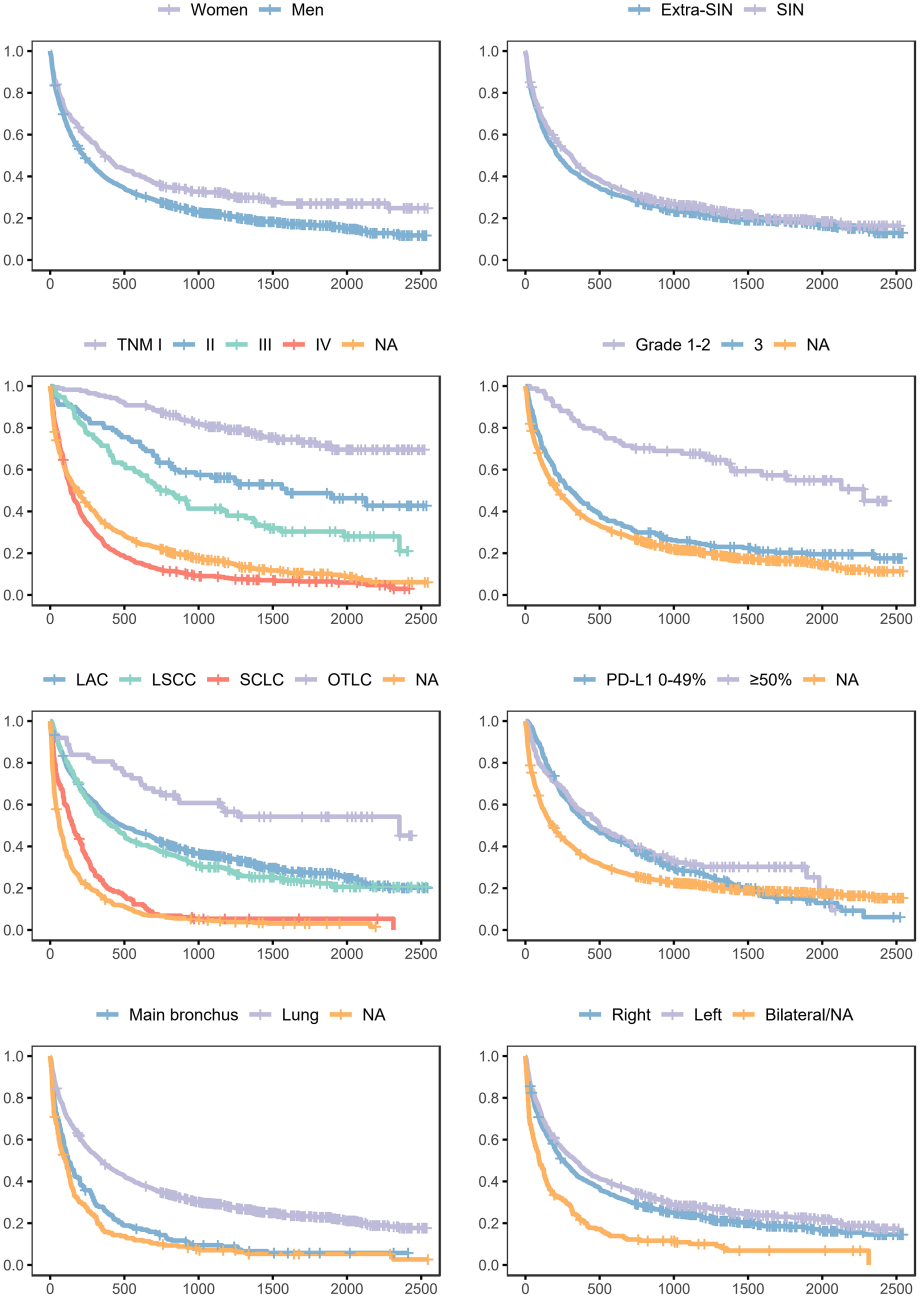
Survival probabilities in the lung cancer cohort, conditional on each analyzed variable and unconditional on other variables. Province of Taranto, 2016–20, follow-up to 31/12/2022. Time: days of follow-up. Outcome (incidence): all-cause death.

Survival probabilities conditional on each analyzed variable and unconditional on other variables are shown in Figure 2. The curves suggested unconditional associations between survival probability and gender, TNM staging, histological grading, morphology, immune checkpoint ligand expression, topography, and laterality. The results of the mixed effects Bayesian Cox proportional hazard regression model are reported in Table 3. Mutually adjusting and adjusting for baseline age class, year, patient ID, and municipality of residence, the fixed effects variables associated with higher death rate were male gender (HR 1.24, 95% CrI 1.07–1.43), TNM II (HR 2.49, 95% CrI 1.63–3.79), III (HR 3.40, 95% CrI 2.33–4.97), IV (HR 8.21, 95% CrI 5.95–11.34) and NA (HR 5.22, 95% CrI 3.78–7.21), grade 3 (HR 1.80, 95% CrI 1.25–2.59) and NA (HR 1.79, 95% CrI 1.27–2.53), and morphologies LSCC (HR 1.18, 95% CrI 1.00–1.39), SCLC (HR 1.62, 95% CrI 1.31–1.99) and NA (HR 2.00, 95% CrI 1.71–2.33). Mutually adjusting and adjusting for baseline age class, year, patient ID, and municipality of residence, the fixed effects variables associated with lower death rate were morphology OTLC (HR 0.65, 95% CrI 0.44–0.95), PD-L1 ≥ 50% (HR 0.75, 95% CrI 0.59–0.95), lung localization (HR 0.73, 95% CrI 0.62–0.86), and left localization (HR 0.85, 95% CrI 0.75–0.95).

**Table 3:** Results of the mixed effects Bayesian INLA Cox proportional hazard regression model in the lung cancer cohort, mutually adjusted and adjusted for baseline age class, year, patient ID, and municipality of residence.

Mixed effects INLA Cox proportional hazard regression	Lung cancer cohort	
	All-cause death	
	*N* = 1,696; person-years = 2,535; *n* = 1,346	
Fixed effects	HR	95% CrI
*Gender*		
Women	1.00	(ref)
Men	1.24	1.07–1.43
*Residence in SIN*		
Extra-SIN	1.00	(ref)
SIN	0.97	0.87–1.08
*Pathological/clinical staging*		
TNM I	1.00	(ref)
TNM II	2.49	1.63–3.79
TNM III	3.40	2.33–4.97
TNM IV	8.21	5.95–11.34
NA	5.22	3.78–7.21
*Histological grading*		
Grade 1–2	1.00	(ref)
Grade 3	1.80	1.25–2.59
NA	1.79	1.27–2.53
*Morphology*		
LAC	1.00	(ref)
LSCC	1.18	1.00–1.39
SCLC	1.62	1.31–1.99
OTLC	0.65	0.44–0.95
NA	2.00	1.71–2.33
*Immune checkpoint ligand expression*		
PD-L1 0–49%	1.00	(ref)
PD-L1 ≥ 50%	0.75	0.59–0.95
NA	1.07	0.91–1.25
*Topography*		
Main bronchus	1.00	(ref)
Lung	0.73	0.62–0.86
NA	0.89	0.70–1.12
*Laterality*		
Right	1.00	(ref)
Left	0.85	0.75–0.95
Bilateral/NA	1.04	0.85–1.27

Province of Taranto, 2016–20, follow-up to 31/12/2022. Time: days of follow-up. Outcome (incidence): all-cause death. N: incident cases and non-cases. n: incident cases.

## Discussion

The results of the present study confirmed that TNM staging, histological grading, morphology, immune checkpoint ligand expression, topography, and laterality are independent prognostic factors for mortality in lung cancer patients. Of interest was the finding during the follow-up period of an overall average negative association between PD-L1 expression and death rate in our cohort, although it may be non-constant over time. This association could be explained by the development and implementation of PD-L1 targeting drugs, such as pembrolizumab, in recent years (4, 6, 7). Pembrolizumab is a humanized monoclonal antibody that inhibits the interaction between the programmed death protein 1 (PD-1) coinhibitory immune checkpoint expressed on tumor cells and infiltrating immune cells and its ligand, PD-L1 (6). In general, patients are eligible for this first-line immunotherapy treatment if their cancer-tissue sample shows a positive expression for PD-L1 in ≥50% of neoplastic cells (4). For these reasons, the presence of PD-L1 ≥ 50% could be assumed as a proxy for treatment with immune checkpoint inhibitors, which is a piece of information not directly available in the cancer registry. Given this assumption, our findings could be supported by the scientific literature on the improvement of overall survival in the treated patients (4, 6, 7). Another interesting result was the negative prognostic value of the presence of missing data (“NA” category) for some variables in our cohort. This could be partly explained by the fact that patients without information on some variables (e.g., grading) could correspond to poor prognosis patients who, due to a severe condition at the time of diagnosis, were unable to undergo further interventions or investigations.

With regard to gender differences in tumor characteristics, according to our study, there appeared to be a lower prevalence of LAC and a higher prevalence of grade 3 tumors among men. While the former result appears to be consistent with gender differences in lung cancer characteristics reported in the literature (15), the latter result was a peculiar and interesting finding of our study. With regard to gender differences in lung cancer prognosis, an important, related result appears to be the association between male patients and increased all-cause death rate. This finding was observed independently of all other factors analyzed, as the HR was adjusted for the other variables included in the Bayesian mixed effects regression model. Specifically, this indicates that male patients in the lung cancer cohort have an excess relative risk for all-cause mortality of 24% (95% CrI 7–43%) compared to female patients. Male patients also present an excess odds ratio for grade 3 tumors of 145% (95% CrI 35–343%) compared to female patients, which is, in turn, a factor independently associated with an increased all-cause death rate. Besides, male patients in the LAC/LSCC cohort present an excess odds ratio for LSCC of 204% (95% CrI 97–369%) compared to female patients, which is also, in turn, a factor independently associated with increased all-cause death rate.

Probably, these direct and indirect effects of gender on overall survival could explain the relative standardized five-years bronchus/lung cancer survival difference between women (24.5) and men (17.8) observed in Taranto Province in the years 2013–17 (31). Moreover, the excess relative risk for mortality independently associated with male patients (direct effect) not only confirms what was already known in relation to the lower survival reported for men in the general population and among lung cancer patients, but also suggests that this excess relative risk could be different in the cohorts followed in this study (14, 16, 35). In this regard, two epidemiological studies on different lung cancer patients’ cohorts reported excess relative risks respectively of 6% and 14% for mortality in men compared to women (14, 16). Although these differences could be attributable to random error, bias, and/or methodological differences, we could not completely rule out the hypothesis that the excess mortality risk in male patients with lung cancer compared to female patients could be different in the population residing in the province of Taranto.

In general, gender-based differences in women with lung cancer could be observed in terms of exogenous risk factors (tobacco use, second-hand smoke, asbestos, radon, radiation, and infections), endogenous risk factors (estrogen and genetic polymorphism), diagnosis (diagnosis at a younger age and with never-smoking history), and outcome and mortality (superior surgical outcomes, differences in response to therapies and adverse effect rates, and improved survival across stages and histologies) (15). Therefore, our findings could indicate an interaction between gender differences in lung cancer prognosis and disadvantaged and/or polluted external context, which is also linked to the second main analyzed determinant in the present study, namely the environmental pressures.

In this regard, the results of this study did not show a clear association between residence in SIN and prevalence of the above mentioned prognostic factors, and between residence in SIN and all-cause death rate. Briefly, this suggests that among the followed-up lung cancer cohort, patients who resided in SIN were supposed to have approximately the same risk of all-cause mortality compared to the patients who resided in other municipalities of the province. To evaluate how the random effect variable municipality of residence affects the association between residence and mortality, in sensitivity analysis, the model was refitted without random effects. Even in this analysis, residence in SIN was not associated with a higher death rate in patients with lung cancer (HR 0.97, 95% CrI 0.87–1.08).

These results do not seem to be consistent with what is already known in relation to the increased risk for all-cause mortality reported for women and men residing in the SIN of Taranto. In fact, the latest epidemiological studies on the resident population reported an excess relative risk for all-cause mortality of 7% (90% CI 5–9%) in women and 10% (90% CI 8–13%) in men in SIN of Taranto compared to the Apulia region for the years 2013–17 (33). A probable explanation is related to the aforementioned very low overall survival of patients with diagnosed invasive lung cancer (31). Specifically, on the one hand, we suppose that the high absolute case fatality rate in these patients is probably not significantly influenced by environmental pressures once the lung cancer has developed, and therefore, it was observed independently from their residence in SIN. On the other hand, we suggest that this high mortality rate in the lung cancer cohort could basically act as an important competing risk to the other causes of death associated with environmental pressures (e.g., cardiovascular diseases) and mask with its magnitude the excess relative risk for all-cause mortality that has been conversely reported for the general population who reside in SIN (31, 33). Besides, a lung cancer cohort is presumably largely made up of smokers or ex-smokers, and tobacco use increases mortality as well. Therefore, the selection of the cohort conditional on the diagnosis of lung cancer could also have influenced the results, preventing the adverse effect of residence in SIN on mortality from being clearly observed.

Whatever the explanations, these findings confirmed the well-known ethical questions regarding the environmental health issues in the contaminated site, as several epidemiological studies have reported an increased risk for lung cancer incidence, hospitalization, and mortality in the entire population residing in the SIN of Taranto (20, 23, 30, 31, 33, 34). In other words, even if we have not found in the present study a difference in survival related to residing in SIN in patients with diagnosed lung cancers, the development of the disease has been clearly associated with residence in SIN in the entire population. In particular, the latest epidemiological data on resident populations reported in SIN an excess relative risk (reference: Taranto Province) for lung cancer incidence of 91% (90% CI 66–120%) in women and 26% (90% CI 16–36%) in men for the years 2015–19 (31). These data raise another point of reflection. Even if female lung cancer patients present a lower all-cause death rate compared to male patients, and even if the women in SIN present a lower absolute incidence rate for lung cancer compared to men, in the SIN of Taranto, a higher excess relative risk for lung cancer incidence was reported in women compared to men (31). This could explain why, in SIN, a higher excess relative risk (reference: Apulia Region) for lung cancer mortality in the general population for the years 2013–2017 was reported in women compared to men (25% vs. 18%) (33). As discussed above, women in our LAC/LSCC cohort also presented a higher prevalence of LAC compared to men. In this regard, it is worth pointing out the interesting finding of the seemingly higher prevalence of LAC in SIN compared to other municipalities, even if the non-effect is included in the 95% credible interval (OR 1.25, 95% CrI 0.94–1.64; given the nature of the data and models, the LAC odds ratio is the reciprocal of the LSCC odds ratio reported in Table 2). The same result was observed in the model without random effects. According to a previous meta-analysis (41), the association with particulate matter exposure was significant for LAC incidence and unclear for LSCC incidence. For these reasons, a hypothesis could be that an overall average higher population exposure to environmental pollutants in SIN could be linked to a higher prevalence of LAC.

However, since this study used an ecological variable (i.e., residence in the SIN of Taranto) to ascertain exposure to environmental pressures, this approach is potentially prone to ecological fallacy. In addition, there is a lack of specificity in the exposure assessment as the specific chemical pollutants could be varied and come from different sources in the studied region. Moreover, in addition to gender differences and the well-known pressures of a strictly environmental nature, the two municipalities of Taranto and Statte present relatively high municipality-level deprivation indexes. This metric is a regionally referenced deprivation index that uses individual data of the general population and housing census of 2011. For the calculation of the index, five conditions were chosen from the authors to best describe the multidimensional concept of social and material deprivation: low level of education, being unemployed, living on rent, living in a crowded house, and living in a single-parent family. The index was calculated as the sum of standardized indicators and is also available categorized into quintiles (42). Although the ecological-level adjustment for the deprivation index was somehow provided by including the municipality of residence in the regression models as a random effect, taking the value of the index itself into consideration with descriptive purposes can also be useful to interpret the results. Regardless, particular attention should be paid to the interpretation of this index due to its ecological-level indicator nature and because the latest available index relates to the 2011 census (42). These limits, therefore, do not guarantee that the available deprivation index represents an accurate indicator of deprivation at the individual level in the years covered by the present study.

However, it should be taken into account that gender, socioeconomic status, deprivation, and inequalities could not only exert an effect on harmful habits (e.g., cigarette smoking), health conditions, and mortality but also potentially affect the utilization of health services (42, 43). Furthermore, in this regard, the SIN corresponds almost completely to the provincial capital, Taranto, which could potentially influence access to health at territorial and hospital levels, and in terms of regional and extra-regional mobility. This is linked to another limitation of the study, which is the lack of available data about the preventive or diagnostic-therapeutic pathways followed by these patients, including information regarding their access to smoking cessation programs/services. In general, the fact that both SIN and extra-SIN municipalities belong to the same Local Health Authority, and so consequentially, the entire studied cohort could virtually access the same healthcare services, did not lead us to suppose that there could be relevant biases in relation to these aspects. However, there is the possibility that residing in the provincial capital could facilitate early cancer diagnosis due to the higher accessibility of the population to healthcare services. In the same way, residing in SIN and being conscious of the overall average higher lung cancer incidence and mortality could potentially influence access to health care services. Conversely, SIN also corresponds to an area with a high level of deprivation, a factor that could potentially exert negative effects on early diagnosis probability, therefore acting in the opposite direction with unclear overall net effects. Socioeconomic deprivation could increase the probability of tobacco use as well. In this regard, another potential limitation of the study could be the lack of information about harmful habits such as smoking or alcohol consumption. These data are unfortunately not available in cancer registries or in health records, and many other published longitudinal studies that use this kind of data lack these details (42–47). However, part of the influence of these factors may have been indirectly captured in the analysis using mixed models with random effects, which take into account the heterogeneity between patients and areas. Moreover, we expected this lack of information to not be a limitation in the strict sense, as these variables could not act as confounders, but rather as mediators between gender and residence in SIN and mortality. In a broad sense, deprivation and tobacco use could be part of a broad range of possible mediators between these factors and mortality, which could comprise socio-cultural factors, risky behaviors, diseases and treatments, and biological factors.

To summarize, as mentioned previously, the lack of information about individual-level environmental exposures, socio-cultural indicators, harmful habits, and utilization of healthcare services could represent a limitation of the present study. However, from another perspective, the same elements could also be considered starting points for what can be done in the future. Specifically, it would be interesting to update and expand upon this epidemiological study by recovering further individual-level data about specific environmental exposures (distance from the different polluting sources, exposure to airborne pollutants through dispersion models, and biomonitoring), risky behaviors (cigarette smoking, alcohol abuse, high-fat diet, and physical inactivity), gender-specific pressures and socioeconomic factors (updated indicators of deprivation at individual or census-tract level), and access to prevention programs and diagnostic-therapeutic paths (timing, place, and type of interventions).

In conclusion, the results confirmed the independent prognostic values of different lung cancer characteristics. Despite the limitations discussed above, even after adjusting for patients and disease characteristics, in the cohort of patients with invasive lung cancer, male gender appeared to be associated with a higher prevalence of poorly differentiated cancer and squamous-cell carcinoma, and with an increased death rate.

## Data availability statement

The original contributions presented in the study are included in the article/supplementary material, further inquiries can be directed to the corresponding author.

## Ethics statement

Ethical approval was not required for the study involving humans in accordance with the local legislation and institutional requirements. Written informed consent to participate in this study was not required from the participants or the participants’ legal guardians/next of kin in accordance with the national legislation and the institutional requirements.

## Author contributions

AM: Data curation, Funding acquisition, Project Administration, Resources, Supervision, Writing – review & editing. SC: Conceptualization, Data curation, Investigation, Methodology, Supervision, Validation, Writing – review & editing. CG: Data curation, Writing - review & editing. MT: Data curation, Writing – review & editing. GML: Data curation, Writing – review & editing. AB: Data curation, Writing – review & editing. IR: Data curation, Methodology, Software, Validation, Writing – review & editing. LB: Data Curation, Resources, Writing – review & editing. RS: Funding acquisition, Methodology, Validation, Writing – review & editing. FA: Investigation, Validation, Writing – review & editing. SM: Data curation, Funding acquisition, Project administration, Resources, Supervision, Writing – review & editing. OVG: Conceptualization, Data curation, Formal analysis, Investigation, Methodology, Project administration, Supervision, Validation, Visualization, Writing – original draft, Writing – review & editing.

## References

[1] [WHO] World Health Organization. Cancer. (2022). Available at: https://www.who.int/news-room/fact-sheets/detail/cancer

[2] Sung H Ferlay J Siegel RL Laversanne M Soerjomataram I Jemal A. Global Cancer statistics 2020: GLOBOCAN estimates of incidence and mortality worldwide for 36 cancers in 185 countries. CA Cancer J Clin. (2021) 71:209–49. doi: 10.3322/caac.21660, PMID: 33538338

[3] [NCI] National Cancer Institute. Lung Cancer Prevention (PDQ®) – Health Professional Version. (2023). Available at: https://www.cancer.gov/types/lung/hp/lung-prevention-pdq (Accessed June 29, 2023).

[4] [AIOM] Italian association of medical oncology. Guidelines. Lung Neoplasms. (2021). Available at: https://www.aiom.it/linee-guida-aiom-2021-neoplasie-del-polmone/ (Accessed June 29, 2023).

[5] Yasukawa M Sawabata N Kawaguchi T Kawai N Nakai T Ohbayashi C. Histological grade: analysis of prognosis of non-small cell lung Cancer after complete resection. In Vivo. (2018) 32:1505–12. doi: 10.21873/invivo.11407, PMID: 30348709 PMC6365755

[6] [NCI] National Cancer Institute. Non-Small Cell Lung Cancer Treatment (PDQ®)–Health Professional Version. (2023). Available at: https://www.cancer.gov/types/lung/hp/non-small-cell-lung-treatment-pdq (Accessed June 29, 2023).

[7] [NCI] National Cancer Institute. Small Cell Lung Cancer Treatment (PDQ®)–Health Professional Version. (2023). Available at: https://www.cancer.gov/types/lung/hp/small-cell-lung-treatment-pdq (Accessed June 29, 2023).

[8] Yang L Wang S Gerber DE Zhou Y Xu F Liu J. Main bronchus location is a predictor for metastasis and prognosis in lung adenocarcinoma: a large cohort analysis. Lung Cancer. (2018) 120:22–6. doi: 10.1016/j.lungcan.2018.03.011, PMID: 29748011 PMC7678407

[9] McWilliam A Vasquez Osorio E Faivre-Finn C van Herk M. Influence of tumour laterality on patient survival in non-small cell lung cancer after radiotherapy. Radiother Oncol. (2019) 137:71–6. doi: 10.1016/j.radonc.2019.04.022, PMID: 31078940

[10] [NCI] National Cancer Institute. NCI Dictionary of Cancer Terms. TNM staging system. (n.d.). Available at: https://www.cancer.gov/publications/dictionaries/cancer-terms/def/tnm-staging-system (Accessed June 29, 2023).

[11] [NCI] National Cancer Institute. Cancer Staging. (2022). Available at: https://www.cancer.gov/about-cancer/diagnosis-staging/staging (Accessed June 29, 2023).

[12] Feng SH Yang ST. The new 8th TNM staging system of lung cancer and its potential imaging interpretation pitfalls and limitations with CT image demonstrations. Diagn Interv Radiol. (2019) 25:270–9. doi: 10.5152/dir.2019.18458, PMID: 31295144 PMC6622436

[13] [NCI] National Cancer Institute. Code for Histologic grading and differentiation. (n.d.). Available at: https://training.seer.cancer.gov/coding/guidelines/rule_g.html (Accessed June 29, 2023).

[14] Yu XQ Yap ML Cheng ES Ngo PJ Vaneckova P Karikios D. Evaluating Prognistic Factors for Sex Differences in Lung Cancer Survival: Findings from a Large Australian Cohort. J Thorac Oncol. (2022) 17:688–99. doi: 10.1016/j.jtho.2022.01.016 35124253

[15] Ragavan M Patel MI. The evolving landscape of sex-based differences in lung cancer: a distinct disease in women. Eur Respir Rev. (2022) 31:210100. doi: 10.1183/16000617.0100-2021, PMID: 35022255 PMC9488944

[16] Sagerup CM Småstuen M Johannesen TB Helland Å Brustugun OT. Sex-specific trends in lung cancer incidence and survival: a population study of 40,118 cases. Thorax. (2011) 66:301–7. doi: 10.1136/thx.2010.151621, PMID: 21199818

[17] [IARC] International Agency for Research on Cancer. Outdoor Air Pollution. IARC monographs on the evaluation of carcinogenic risks to humans; volume 109. (2015). Available at: https://publications.iarc.fr/Book-And-Report-Series/Iarc-Monographs-On-The-Identification-Of-Carcinogenic-Hazards-To-Humans/Outdoor-Air-Pollution-2015 (Accessed June 26, 2023).

[18] [EEA] European Environment Agency. Air pollution. (2022). Available at: https://www.eea.europa.eu/publications/environmental-burden-of-cancer/air-pollution

[19] Chen J Hoek G. Long-term exposure to PM and all-cause and cause-specific mortality: a systematic review and meta-analysis. Environ Int. (2020) 143:105974. doi: 10.1016/j.envint.2020.105974, PMID: 32703584

[20] Alessandrini ER Leogrande S Morabito A Ancona C Assennato G Giua R. Studio di coorte sugli effetti delle esposizioni ambientali ed occupazionali sulla morbosità e mortalità della popolazione residente a Taranto. (2016). Available at: https://www.sanita.puglia.it/documents/890301/896208/Relazione+Finale+Studio+di+Coorte+-+2016/ea231c81-e196-4b43-99a4-0882bd60b83b (Accessed November 5, 2023).

[21] Bustaffa E Minichilli F Andreassi MG Carone S Coi A Cori L. Studi su marcatori di esposizione ed effetto precoce in aree con inquinamento da arsenico: metodi e risultati del progetto SEpiAs. Sorveglianza epidemiologica in aree interessate da inquinamento ambientale da arsenico di origine naturale o antropica (SEpiAS CCM 2010) [studies on markers of exposure and early effect in areas with arsenic pollution: methods and results of the project SEpiAs. Epidemiological surveillance in areas with environmental pollution by natural or anthropogenic arsenic]. Epidemiol Prev. (2014) 38:27–94. PMID: 25115552

[22] Comba P Pirastu R Conti S De Santis M Iavarone I Marsili G. Ambiente e salute a Taranto: studi epidemiologici e indicazioni di sanità pubblica [environment and health in Taranto, Southern Italy: epidemiological studies and public health recommendations]. Epidemiol Prev. (2012) 36:305–20. PMID: 23293255

[23] Galise I Serinelli M Morabito A Pastore T Tanzarella A Laghezza V. L’impatto ambientale e sanitario delle emissioni dell’impianto siderurgico di Taranto e della centrale termoelettrica di Brindisi [The integrated environmental health impact of emissions from a steel plant in Taranto and from a power plant in Brindisi, (Apulia region, Southern Italy)]. Epidemiol Prev. (2019) 43:329–37. doi: 10.19191/EP19.5-6.P329.102, PMID: 31659880

[24] Giannico OV Baldacci S Basile FC Pellegrino A Desiante F Franco E. PCDD/Fs and PCBs in hen eggs from a contaminated area in Italy: a 9 years spatio-temporal monitoring study. Food Addit Contam Part A Chem Anal Control Expo Risk Assess. (2023) 40:294–04. doi: 10.1080/19440049.2022.2157051, PMID: 36602427

[25] Giannico OV Baldacci S Desiante F Basile FC Franco E Fragnelli GR. PCDD/Fs and PCBs in *Mytilus galloprovincialis* from a contaminated area in Italy: the role of mussel size, temperature and meteorological factors. Food Addit Contam Part A Chem Anal Control Expo Risk Assess. (2022) 39:1123–35. doi: 10.1080/19440049.2022.2059108, PMID: 35389328

[26] Giannico OV Fragnelli GR Baldacci S Desiante F Pellegrino A Basile FC. Dioxins and PCBs contamination in milk and dairy products from province of Taranto (Puglia Region, Southern Italy): a six years spatio-temporal monitoring study. Ann Ist Super Sanita. (2021) 57:233–8. doi: 10.4415/ANN_21_03_06, PMID: 34554117

[27] Giannico OV Desiante F Basile FC Franco E Baldacci S Fragnelli GR. Dioxins and PCBs contamination in mussels from Taranto (Ionian Sea, Southern Italy): a seven years spatio-temporal monitoring study. Ann Ist Super Sanita. (2020) 56:452–61. doi: 10.4415/ANN_20_04_07, PMID: 33346171

[28] Iavarone I De Felip E Ingelido AM Iacovella N Abballe A Valentini S. Exploratory biomonitoring study among workers of livestock farms of the Taranto Province. Epidemiol Prev. (2012) 36:321–31. PMID: 23293256

[29] Ingelido AM Abate V Abballe A Albano FL Battista T Carraro V. Concentrations of polychlorinated dibenzodioxins, polychlorodibenzofurans, and polychlorobiphenyls in women of reproductive age in Italy: a human biomonitoring study. Int J Hyg Environ Health. (2017) 220:378–86. doi: 10.1016/j.ijheh.2016.11.009, PMID: 27908667

[30] Leogrande S Alessandrini ER Stafoggia M Morabito A Nocioni A Ancona C. Industrial air pollution and mortality in the Taranto area, Southern Italy: a difference-in-differences approach. Environ Int. (2019) 132:105030. doi: 10.1016/j.envint.2019.105030 31398654

[31] Minerba S Mincuzzi A Bruni A Carone S Galluzzo C Lagravinese GM. ASL Taranto, AReSS Puglia. Rapporto sui tumori ASL di Taranto (2021). Available at: https://www.sanita.puglia.it/documents/36057/232392045/2021+Report+Registro+Tumori+-+anni+2015-2019.pdf/3439fd81-2943-49a5-87e2-810ba83326ff (Accessed November 5, 2023).

[32] Pirastu R Comba P Iavarone I Zona A Conti S Minelli G. Environment and health in contaminated sites: the case of Taranto, Italy. J Environ Public Health. (2013):753719. doi: 10.1155/2013/753719, PMID: 24454414 PMC3886576

[33] Zona A Fazzo L Benedetti M Bruno C Vecchi S Pasetto R. SENTIERI - Studio epidemiologico nazionale dei territori e degli insediamenti esposti a rischio da inquinamento. Sesto Rapporto [SENTIERI - Epidemiological Study of Residents in National Priority Contaminated Sites. Sixth Report]. Epidemiol Prev. (2023) 47:1–286. doi: 10.19191/EP23.1-2-S1.003, PMID: 36825373

[34] Zona A Iavarone I Buzzoni C Conti S Santoro M Fazzo L. SENTIERI: Studio epidemiologico nazionale dei territori e degli insediamenti esposti a rischio da inquinamento. Quinto Rapporto [SENTIERI: Epidemiological Study of Residents in National Priority Contaminated Sites. Fifth Report]. Epidemiol Prev. (2019) 43:1–208. doi: 10.19191/EP19.2-3.S1.032, PMID: 31295974

[35] [ISTAT] National Institute of Statistics. Demo. Demography in numbers. (2023). Available at: https://demo.istat.it/ (Accessed July 5, 2023).

[36] R-INLA. Cox proportional hazards model. (2023). Available at: https://inla.r-inla-download.org/r-inla.org/doc/likelihood/coxph.pdf (Accessed March 25, 2023).

[37] Rue H Riebler A Sørbye SH Illian JB Simpson DP Lindgren FK. Bayesian computing with INLA: a review. Annu Rev Statist Appl. (2017) 4:395–21. doi: 10.1146/annurev-statistics-060116-054045

[38] Rue H Martino S Chopin N. Approximate Bayesian inference for latent gaussian models by using integrated nested Laplace approximations. J R Statis Soc Ser B. (2009) 71:319–92. doi: 10.1111/j.1467-9868.2008.00700.x

[39] Wang X Yue YR Faraway JJ. Bayesian regression Modeling with INLA. 1st ed. Boca Raton: Chapman and Hall/CRC. (2018). doi: 10.1201/9781351165761

[40] Martino S Akerkar R Rue H. Approximate Bayesian inference for survival models. Scand J Stat. (2011) 38:514–28. doi: 10.1111/j.1467-9469.2010.00715.x

[41] Raaschou-Nielsen O Andersen ZJ Beelen R Samoli E Stafoggia M Weinmayr G. Air pollution and lung cancer incidence in 17 European cohorts: prospective analyses from the European study of cohorts for air pollution effects (ESCAPE). Lancet Oncol. (2013) 14:813–22. doi: 10.1016/S1470-2045(13)70279-1, PMID: 23849838

[42] Rosano A Pacelli B Zengarini N Costa G Cislaghi C Caranci N. Aggiornamento e revisione dell’indice di deprivazione italiano 2011 a livello di sezione di censimento [Update and review of the 2011 Italian deprivation index calculated at the census section level]. Epidemiol Prev. (2020) 44:162–70. doi: 10.19191/EP20.2-3.P162.039, PMID: 32631016

[43] Carstairs V. Deprivation indices: their interpretation and use in relation to health. J Epidemiol Community Health. (1995) 49:S3–8. doi: 10.1136/jech.49.suppl_2.s3, PMID: 8594130 PMC1060868

[44] Allemani C Matsuda T Di Carlo V Harewood R Matz M Nikšić M. Global surveillance of trends in cancer survival 2000-14 (CONCORD-3): analysis of individual records for 37 513 025 patients diagnosed with one of 18 cancers from 322 population-based registries in 71 countries. Lancet. (2018) 391:1023–75. doi: 10.1016/S0140-6736(17)33326-3, PMID: 29395269 PMC5879496

[45] Dal Maso L Panato C Guzzinati S Serraino D Francisci S Botta L. Prognosis and cure of long-term cancer survivors: a population-based estimation. Cancer Med. (2019) 8:4497–507. doi: 10.1002/cam4.2276, PMID: 31207165 PMC6675712

[46] Coviello V Buzzoni C Fusco M Barchielli A Cuccaro F De Angelis R. Survival of cancer patients in Italy. Epidemiol Prev. (2017) 41:1–244. doi: 10.19191/EP17.2S1.P001.017, PMID: 28629213

[47] Giannico OV Carone S Tanzarella M Galluzzo C Bruni A Lagravinese GM. Environmental pressures, tumor characteristics, and death rate in a female breast cancer cohort: a seven-years Bayesian survival analysis using cancer registry data from a contaminated area in Italy. Front Public Health. (2023) 11:1310823. doi: 10.3389/fpubh.2023.1310823 PMC10805021 38264246

